# Exercise Intensity and Validity of the Ratings of Perceived Exertion (Borg and OMNI Scales) in an Indoor Cycling Session

**DOI:** 10.2478/hukin-2013-0072

**Published:** 2013-12-31

**Authors:** José M. Muyor

**Affiliations:** 1 Faculty of Education Sciences, Nursing and Physiotherapy. Laboratory of Kinesiology, Biomechanics and Ergonomics (KIBIOMER Lab), University of Almería, Almería. SPAIN

**Keywords:** Spinning®, wellness, aerobic, effort, cardiovascular, fitness

## Abstract

The purpose of the study was: 1) to determine the intensity of an indoor cycling session; 2) to know the correlation between the rating of perceived exertion (RPE) scales (Borg and OMNI) and % heart rate reserve (%HRR) with categories; and 3) to evaluate the validity of RPE scales (Borg and OMNI) with respect to the heart rate (HR) and %HRR. A total of fifty-three subjects, 25 males and 28 females (ages: 28.79 ± 6.04 years; body height: 1.71 ± 0.09 m; body mass: 69.59 ± 13.69 kg) were recruited from a private fitness club. All subjects performed the same predesigned indoor cycling session with a total duration of 50 minutes. During the experimental trial, the HR was recorded every 5 s. The Borg 6–20 RPE and OMNI 0–10 scales were used to assess perceived exertion in each phase. The average HR in the cardiovascular phase was 152.24 ± 14.11 b•min-1, the %HRR was 80.62 ± 7.10; and the overall RPE (Borg and OMNI scales) was 14.94 ± 1.11 and 7.18 ± 0.79 points, respectively. The correlation between an average HR and %HRR with Borg and OMNI scales was lower than r = 0.4 (p < 0.05). The correlation value between the Borg and the OMNI RPE scales was r = 0.82 (p < 0.001). It can be concluded that indoor cycling elicits effort of high intensity which could be inappropriate for some participants. The Borg and OMNI scales showed a low validity to quantify the intensity performed in indoor cycling sessions. It indicates the necessity to control the intensity of effort with other instruments to improve efficacy and decrease the risk of overload in this activity.

## Introduction

Nowadays, indoor cycling, also known as Spinning® (registered trademark of Madd Dog Athletics, Inc) is an activity offered in most fitness centers. It is an activity where participants, normally of different ages, body mass index, fitness condition and cardiorespiratory capacity cycle together on a specific stationary bicycle, following the rhythm of music and the instructions of an indoor cycling trainer to perform the geographic virtual profile programmed. The indoor cycling trainer orders an intensity to reach in each music track and the participants have to adjust the tension on the flywheel.

Indoor cycling classes are usually very demanding because this activity is viewed as an exercise where a large number of calories is burned. Therefore, a lot of sedentary people participate in this activity in order to lose weight; it is a motivating activity for them. In this sense, Valle et al. (2009; 2010) reported that indoor cycling associated with a restricted diet is an excellent option in controlling obesity and serum lipids. Bianco et al. (2010) found a decrease in body weight, without any restriction on food consumption, and an improvement in cardiorespiratory fitness in young overweight women. However, these authors fundamentally recommend training protocols which are intense and length specific to the fitness level of the participants.

Several studies have reported that indoor cycling is a strenuous physical activity (Battista et al., 2008; Caria et al., 2007; Foster et al., 2006; López-Minarro and Muyor, 2010; Muyor and López-Miñarrro, 2012; Richey et al., 1999) which may be inappropriate for novice subjects (Battista et al., 2008; Caria et al., 2007; Foster et al., 2006). Moreover, in literature there are some rhabdomyolysis cases due to the practice of indoor cycling (Montero et al., 2009; Young and Thompson, 2004). Battista et al. (2008) found in two simulated indoor cycling classes that there are some moments of the session when the VO2 exceeded the VO2max observed during incremental testing. Crumpton et al. (1999) found that the HR response was 83% of the subjects’ age-predicted maximum in 40 minutes of indoor cycling. Kang et al. (2005) found that a Spinning® (indoor cycling) session resulted in a greater VO2 in comparison to a constant intensity protocol in cycling. López-Miñarro and Muyor (2010) found mean values of the % heart rate reserve (%HRR) around 72% in the cardiovascular phase in novice subjects. Recently, Muyor and López-Miñarro (2012) found a %HRR of around 80% in subjects who had 6 months experience in the indoor cycling.

Most fitness centers do not have a physiological department and biomedical instruments such as an electrocardiogram or VO2 equipment to evaluate the physiological responses in their users because it is quite expensive and requires qualified staff. For these reasons, it is common to use a heart rate monitor or rating of perceived exertion (RPE) scales. The RPE is a recognized marker of intensity and of homeostatic disturbance during exercise (Eston, 2012). In fact, previous studies have shown strong relationships between RPE and physiological parameters such as blood lactate response (Irving et al., 2006) and VO2 (Eston et al., 2006; Eston et al., 2005). Other studies have evaluated the validity and reliability of RPE for elite swimmers (Psycharakis, 2011), Australian footballers (Scott et al., 2013), trained male runners (Coquart and Garcin, 2007), and cyclists (Pérez-Landaluce et al., 2002; Shigematsu et al., 2004). All these studies reported that the RPE is of practical value to prescribe exercise training intensities.

Also, the RPE’s validity has been evaluated to control the intensity in aerobic resistance activities performed in fitness centers, such as bench stepping exercise (Ozkan and Kin-Isler, 2007). In this case, Ozkan and Kin-Isler (2007) found that the RPE was a reliable but not a valid method for regulating exercise intensity in step dance sessions. Other studies have used the RPE to measure intensity in aerobic dance sessions (Laukkanen et al., 2001) or specifically, in indoor cycling sessions (Battista et al., 2008; Crumpton et al., 1999). These studies did not evaluate the validity of the RPE while the sessions were performed. López-Miñarro and Muyor (2010) analyzed the validity of the RPE in novice subjects who had an experience in indoor cycling of fewer than 12 weeks. They found a reduced validity for the overall RPE (r = 0.41, p < 0.05). Recently, Muyor and López-Miñarro (2012) did not find a good validity of the RPE (r = 0.18, p > 0.05) in subjects who had between 24 and 28 weeks (2–3 classes per week) of experience in indoor cycling. In these studies the overall RPE was measured after the session and they only used the Borg 6–20 RPE scale.

Due to the popularity of indoor cycling in fitness centers, and because it has been classified as a high intensity activity, as well as taking into account the lack of control of effort by the participants, the objectives of this study were as follows: 1) to determine the intensity of the indoor cycling session following the ACSM categories; 2) to define the correlation between the RPE scales (Borg and OMNI) and the %HRR categories; and 3) to evaluate the validity of the RPE scales (Borg and OMNI) with respect to HR and %HRR.

## Material and Methods

### Participants

A total of fifty-three healthy subjects, 25 males and 28 females (age: 28.79 ± 6.04 years; body height: 1.71 ± 0.09 m; body mass: 69.59 ± 13.69 kg) were recruited from a private fitness club and voluntarily participated in the study. All were experienced in indoor cycling, and all had been participating in indoor cycling at least three days per week for the preceding 3 months at the time of the study. None of the subjects performed exhausting efforts and all abstained from caffeine and stimulating drinks at least 48h prior to the study. Each subject provided written informed consent before the measurements were taken. The study was approved by the Institutional Review Board at the University of Almería (Spain).

### Procedures

The indoor cycling session was carried out between 19:00 h and 21:00 h. Room temperature and relative humidity were 21.0 ± 2.5 Cº and 46.0 ± 6.5%, respectively, and friction-loaded specific bicycles for indoor cycling were used (BH^®^, BH Duke^®^, Spain). All subjects performed the same predesigned indoor cycling session with a total duration of 50 minutes. The subjects were fitted with a chest HR transmitter and wrist monitor recorder. HR was recorded, from the beginning of the session, using individual Polar RS400 (Polar^®^ Vantage NV, Polar Electro Oy, Finland), and subsequently exported and analyzed using the Polar Pro-Trainer^®^ software program (Polar Electro Oy, Finland).

The subjects could not see their HR measurements during the experimental trial, because it could influence their perceived effort on the Borg and OMNI RPE scales. For this reason, a sticker was placed on each HR monitor.

The experimental trial was divided into four stages: a warm-up (10 minutes in a seated position, with a cadence of 90–100 RPM (revolutions per minute)), a main phase (35 minutes, where the subjects alternated between normal seated positions and seated and standing climb cycling, between 60–80 RPM in climb techniques and between 80 – 110 RPM in normal seated cycling). Then, a cool down (5 minutes, with a cadence of 80–100 RPM) in a seated position and, finally, stretching exercises, of the principal muscles used in the session off cycling.

During the experimental trial, HR was recorded every 5 s. The participants were instructed to follow the directions of a qualified indoor cycling instructor, which included recommended frequencies of pedalling (RPM) in each phase of the session and recommended cycle resistance. The instructor provided feedback to help the subjects to regulate their intensity. Although the resistance of the cycle could be freely changed by the participants during the session, the study subjects had to follow the instructions about the resistance and the RPM indicated by the instructor.

The Borg 6–20 RPE and the OMNI 0–10 scales were used to assess perceived exertion. The RPE is a 15-point single-item scale ranging from 6 to 20, with anchors ranging from 6 “No exertion” to 20 “Maximum exertion”. The OMNI 0–10 scale has a category rating format that contains both pictorial and verbal descriptors positioned along a comparatively narrow numerical response range, 0–10. Each pictorial descriptor is consistent with its corresponding verbal descriptor, from 0 “Extremely easy” to 10 “Extremely hard”. Both RPE scales were positioned within sight in the indoor cycling room. The subjects were instructed to give an overall perception about how hard the exercise felt according to both RPE scales every five minutes, from the start to the end of the indoor cycling session. These values were written on a record sheet which the subjects had on their handlebars. Before the measurements, subjects were asked to read instructions on how to use these scales.

A familiarization period of two weeks (and a minimum of 3 sessions per week) prior to the experimental trial was carried out to accustom the participants with the Borg and the OMNI RPE scales. The first session consisted of familiarization to the RPE scales. Each subject was given a copy of the RPE scales for their use during the exercise sessions. The subjects were instructed to read the scale before each session, to create an awareness of their exercise stimulus range and the possible RPE responses.

Maximum HR was predicted from the 220 – age formula if the subjects were under forty years old and the 206.9 – (0.67 × age) formula if they were older than 40 years (Gellish et al., 2007). Later, the percentage of heart rate reserve (%HRR) was calculated for each subject. Heart rate reserve (HRR) was determined by predicted maximum HR minus resting HR. The HRR percentage was determined by (exercise HR – resting HR) X 100/HRR. It is the percentage of the difference between resting and maximal HR. The intensity category was determined using the American College of Sports Medicine classification (Table 1).

### Statistical Analyses

Means and standard deviations (SD) were calculated for all variables. A dependent *t*-test was conducted to determine whether a significant difference exists between HR resting and HR in the end of the cool down (after stretching exercises). Mean values of %HRR, Borg RPE and OMNI RPE scales, every five minutes during the indoor cycling session, were plotted. Distributions of subjects among categories of exercise intensity were examined using the Chi^2^ test. Contingency table analyses were used to assess the association between Borg and OMNI RPE scales and %HRR categories of intensities. The relationship between HR and %HRR (criterion measures) and both RPE scales recorded (Borg and OMNI scales), in the main phase, were determined using the Pearson product-moment correlation coefficient to determine the validity of both the RPE scales with respect to mean HR and %HRR (criterion measure). Statistical significance was set at *p* < 0.05. Analyses were performed using the SPSS 18.0 statistical software package.

## Results

The mean and standard deviation values of heart rate (HR) and percentage of heart rate reserve (%HRR) at rest, main phase, at the end of cool down, and in the total session (mean HR warm up + mean HR main phase + mean HR cool down / 3), as well as mean rating of perceived exertion (RPE) values of the Borg and the OMNI scales during the main phase and the total session are presented in Table 2.

The mean maximum HR in the main phase was 176.91 ± 11.02 b·min^−1^. There were significant differences between resting HR and the final cool down HR (31.18 ± 16.39 b·min^−1^; *p* < 0.001) and between % Resting HRR and % final cool down HRR (16.33 ± 8.67; *p* < 0.001).

**Figure 1:**
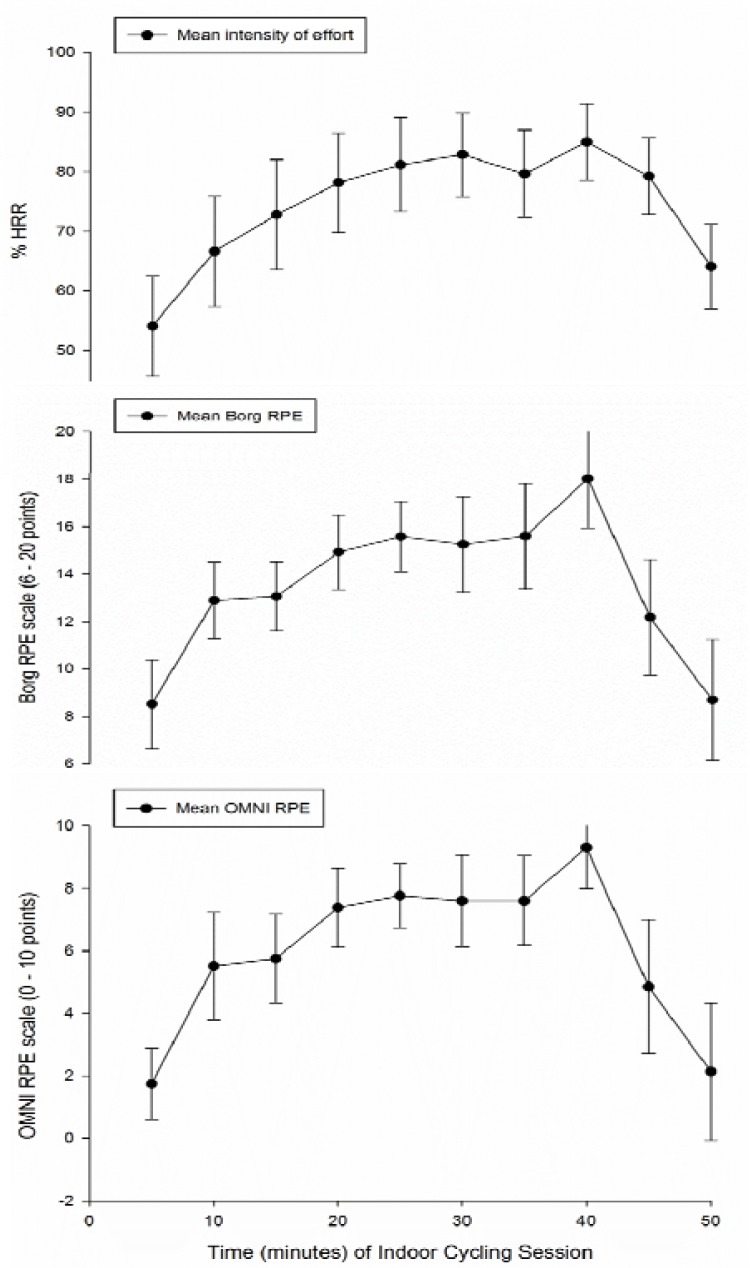
Mean *values of %HRR, the Borg RPE and the OMNI RPE scales, every five minutes during the indoor cycling session*

Mean values of %HRR, the Borg RPE and the OMNI RPE scales, every five minutes during the indoor cycling session, are presented in Figure 1. Mean %HRR was greater than 65% (hard intensity) from the 10 minute mark (after the warm up) until the 45 minute mark (at the end of the main phase). The average RPE values in both scales were greater than 14 points (on the Borg scale) and 7 points (on the OMNI scale) (in both scales “high intensity”) from the 20 minute to the 40 minute mark.

A total of 26 subjects (49.1%) reached similar intensities in both the overall Borg RPE scale and %HRR. Twenty-seven subjects (50.1%) perceived lower exercise intensity than they were truly performed (Table 3). A total of 19 subjects (35.9%) reached similar intensity in both the overall OMNI RPE scale and %HRR. Thirty-four subjects (64.1%) perceived lower exercise intensity than they really performed (Table 3).

There were significant but low correlations (*r* = 0.29; r = 0.27, *p* < 0.001) between the average HR, and Borg, and OMNI RPE scales in the main phase, respectively. The %HRR showed moderate and significant correlation values between the %HRR and Borg, and OMNI RPE scales in the main phase (*r* = 0.37; *r* = 0.31, *p* < 0.001), respectively. The correlation value between the Borg and the OMNI RPE scales was *r* = 0.82 (*p* < 0.001).

## Discussion

In the literature, there are few studies which have evaluated the intensity of effort and the RPE validity during real indoor cycling sessions. The main purpose of this study was to determine the intensity of the indoor cycling session following the ACSM′s categories in a real setting (private fitness club); to define the correlation between the RPE scales (Borg and OMNI) and %HRR categories and to evaluate the validity RPE scales (Borg and OMNI) with respect to HR and %HRR.

The results show that indoor cycling is a demanding activity. In the main phase, the average %HRR, RPE Borg and RPE OMNI were 80.62 ± 7.10 %; 14.94 ± 1.11 points and 7.18 ±0.79 points, respectively. All these data correspond to high intensity following the ACSM guidelines. These findings are in agreement with previous studies performed in indoor cycling (Battista et al., 2008; Caria et al., 2007; Foster et al., 2006; López-Minarro and Muyor, 2010; Montero et al., 2009; Muyor and López-Miñarrro, 2012; Richey et al., 1999). Battista et al. (2008) found that the average % VO2max during an indoor cycling class (45 minutes) was in the range of 75% and 80% of VO2max. 52% of the time was spent at an intensity level greater than the ventilatory threshold. The exercise was perceived as quite strenuous (7.6 ± 1.6) in an RPE scale from 0 to 10 points. Caria et al. (2007) found, in Spinning® instructors, that the mean values corresponded to 88% and 85% of their HRmax for male and female instructors, respectively. The higher percentage of effort in that study with respect to the current study might be due to an evaluation of instructors who could be more motivated to reach higher intensity. Foster et al. (2006) reported an average of 89 ± 5 and 83 ± 6 %HRmax and RPE 7.6 ± 0.9 and 6.3 ± 1.2 (from a 0 to 10 scale) in two kinds of typical indoor cycling sessions. Crumpton et al. (1999) found that the HR response was 83% of the subjects′ age-predicted maximum. All these studies were performed in artificial situations because the measurements were taken in laboratories. In a real situation, especially, during a real indoor cycling session in a private fitness center, López-Miñarro and Muyor (2010) reported in novice indoor cycling participants, a cardiovascular phase of around 72%HRR and an RPE 14 points (using the Borg scale from 6 to 20 points). Recently, Muyor and López-Miñarro (2012) found an average of 78%HRR, and RPE 14 points. All the studies mentioned agree that indoor cycling elicits high intensity levels. Moreover, the current study finds that the final HR and %HRR (after the stretching exercises) was higher than before the session (p < 0.001). Therefore, the recovery phase was incomplete. All these reasons would make controlling the intensity during the indoor cycling session necessary.

In line with the aforementioned, another purpose of the current study was to evaluate the validity of RPE scales (Borg and OMNI) with respect to HR and %HRR. In this sense, the results reported that both the Borg and the OMNI scales are not valid to quantify the intensity performed in indoor cycling sessions. Although a high correlation value between the Borg and the OMNI scales (r = 0.82, p < 0.001) was found, the correlation between average HR and %HRR with Borg and OMNI scales was lower than r = 0.4 but significant (p < 0.05). These results are in agreement with previous studies performed in indoor cycling. López-Miñarro and Muyor (2010) found a reduced validity between the Borg RPE scale and %HRR (r = 0.41, p < 0.05) in fifty-nine subjects with limited experience in indoor cycling (between 4 and 12 weeks and 2–3 classes per week). Consequently, Muyor and López-Miñarro (2012) evaluated eighty subjects who had experience between 24 and 28 weeks (2–3 classes per week) in indoor cycling. However, these authors found a low correlation between the Borg RPE scale and %HRR (r = 0.18, p > 0.05). These authors explained their results by the fact that the participants may have had little experience in indoor cycling and because they would not have understood the methodology of the Borg scale. However, in the current study we used two scales, the Borg and the OMNI scales in more experienced subjects than in prior studies but we did not find acceptable validity values in none of these scales.

When the distribution of subjects with regards to intensity categories obtained from the overall RPE in the Borg and the OMNI scales, and %HRR was analyzed, we found that fewer than 50% of the subjects perceived (in the Borg and the OMNI scales) the same intensity than they were performing (calculated with %HRR). However, more than 50% of subjects performed higher intensity levels than they perceived. This discrepancy between the intensity perceived and performed could explain the low validity found in the RPE Borg and OMNI scales.

Several studies have reported that the RPE Borg and OMNI scales are valid to quantify the intensity of effort in several athletes such as elite swimmers, Australian footballers, runners, and cyclists (Coquart and Garcin, 2007; Pérez-Landaluce et al., 2002; Psycharakis, 2011; Scott et al., 2013; Shigematsu et al., 2004). However, the protocols used in these studies were incremental tests. This circumstance could lead to higher values of correlation between HR parameters and RPE scales. In contrast, indoor cycling is characterized for interval profiles. The indoor cycling instructors order an intensity to reach in each music track and the participants have to adjust the tension on the flywheel. Although the instructor gives information to carry out, in most cases the participants do not follow the instructions and they freely adjust the resistance in their bicycle according to the fatigue perceived at each moment. Normally, the participants do not use a heart rate monitor in their sessions. Moreover, in the current study, although the participants had a heart rate monitor to record the heart rate for the evaluation, they could not see it so as not to influence the subjective information given. In indoor cycling, the participants could reduce the resistance in their bicycles when they felt local fatigue in their lower limb muscles, although their heart rate could be high. This could explain why a high percentage of subjects had lower perception of intensity of effort (because they reduced the resistance); however, their %HRR performed was high (because their HR remained high). So, this could help to explain why the RPE Borg and OMNI scales are not valid tools to quantify the intensity of effort in this activity. Future studies could evaluate the validity of RPE scales in indoor cycling, however, controlling that the participants could not freely modify their resistance on the flywheel.

In conclusion, the current study confirmed results reported in previous studies, namely, indoor cycling elicits a high intensity of effort which could be inappropriate in some participants (mainly in new participants or with low physical fitness). Moreover, the low validity found in both the RPE scales (Borg and OMNI) indicates the necessity to control the intensity of effort with other instruments. The current study reports that a high percentage of participants perceived a lower intensity than they were actually performing. This situation could generate some cardiovascular risks. Because the RPE scales (Borg and OMNI) are not valid instruments in this activity, we suggest using a heart rate monitor to control the intensity. Indoor cycling instructors could introduce a table with the intensity zones and heart rates to each participant. In this sense, when the instructor indicates the intensity to reach, each participant could set the adequate resistance in the bicycle following the intensity of effort from the monitored heart rate. Thus, the inadequate intensity perceived would be avoided.

## Figures and Tables

**Table 1:** Classification of physical activity intensity

Intensity	%HRR	Borg RPE	OMNI RPE
Very light	< 20	<10	0 – 1
Light	20 – 39	10 – 11	2
Moderate	40 – 59	12 – 13	3 – 6
High	60 – 84	14 – 16	7 – 8
Very High	≥ 85	17 – 19	9
Maximal	100	20	10

Modified of ACSM Position Stand.

%HRR: percent of heart rate reserve.

Borg RPE: Borg rating of perceived exertion 6–20 Scale.

OMNI RPE: OMNI rating of perceived exertion 0–10 Scale.

**Table 2:** The mean and standard deviation values of HR, %HRR and RPE scales in the indoor cycling session

		Mean ± SD	Intensity category^†^
Resting	HR^*^	79.79 ± 12.35	-
	%HRR	41.74 ± 6.41	Moderate
End of cool down	HR^*^	110.98 ± 13.43	-
	%HRR	58.08 ± 7.15	Moderate
Main Phase	HR^*^	152.24 ± 14.11	-
	%HRR	80.62 ± 7.10	High
	RPE Borg	14.94 ± 1.11	High
	RPE OMNI	7.18 ± 0.79	High
Total Session (warm up + main phase + cool down + stretching exercises)	HR^*^	141.68 ± 13.17	-
	%HRR	74.32 ± 6.72	High
	RPE Borg	15.70 ± 1.50	High
	RPE OMNI	7.98 ± 0.77	High

* HR values are expressed in (b·min^−1^)

† Proposed by the ACSM

**Table 3:** Distribution of subjects with regards to intensity categories obtained from overall ratings of perceived exertion in the Borg and in the OMNI RPE scales and percentage of heart rate reserve (%HRR) during the main phase of the indoor cycling class

		Borg RPE Categories		
		Moderate (12–13 points)	High (14–16 points)	Very High (17–19 points)

%HRR categories	High (60–84%)	17.0%	47.2%	0%
	Very High (85–95%)	3.7%	30.2%	1.9%

		OMNI RPE Categories		
		Moderate (3–6 points)	High (7–8 points)	Very High (9 points)

%HRR categories	High (60–84%)	30.2%	34.0%	0%
	Very High (85–95%)	11.3%	22.6%	1.9%
